# 3D Printing in Regenerative Medicine: Technologies and Resources Utilized

**DOI:** 10.3390/ijms232314621

**Published:** 2022-11-23

**Authors:** Antreas Kantaros

**Affiliations:** Department of Industrial Design and Production Engineering, University of West Attica, 12244 Athens, Greece; akantaros@uniwa.gr

**Keywords:** 3D printing, bio-printing, biomaterials, fused deposition modeling (FDM), stereolithography (SLA), direct ink writing (DIW), laser-guided direct writing (LGDW)

## Abstract

Over the past ten years, the use of additive manufacturing techniques, also known as “3D printing”, has steadily increased in a variety of scientific fields. There are a number of inherent advantages to these fabrication methods over conventional manufacturing due to the way that they work, which is based on the layer-by-layer material-deposition principle. These benefits include the accurate attribution of complex, pre-designed shapes, as well as the use of a variety of innovative raw materials. Its main advantage is the ability to fabricate custom shapes with an interior lattice network connecting them and a porous surface that traditional manufacturing techniques cannot adequately attribute. Such structures are being used for direct implantation into the human body in the biomedical field in areas such as bio-printing, where this potential is being heavily utilized. The fabricated items must be made of biomaterials with the proper mechanical properties, as well as biomaterials that exhibit characteristics such as biocompatibility, bioresorbability, and biodegradability, in order to meet the strict requirements that such procedures impose. The most significant biomaterials used in these techniques are listed in this work, but their advantages and disadvantages are also discussed in relation to the aforementioned properties that are crucial to their use.

## 1. Introduction

Additive manufacturing, more often referred to as “3D printing,” is the method of fabricating three-dimensional objects by adding successive layers of materials at a regulated rate and thickness. These materials could be made of concrete, metals, ceramics, polymers, resins, biomaterials, or other substances. The dearth of variety in 3D-printable materials continues even though printing time, processing speed, and printing resolution have all increased over the past few years. The compatibility and flowability of printing ink with the current printing procedures are crucial for developing fields such as the 3D printing of biomaterials, tissues, and high-viability cells. This study details the improvements in 3D printing materials for new biomedical applications and how well they work with already-used printing technologies.

In the additive process of bio-printing, cells and biomaterials are sequentially deposited to create cell patterns, creating living human constructions with comparable biological, chemical, and mechanical properties for optimal tissue, scaffold, and organ recovery. The substances used in this procedure are referred to as biomaterials, and they should have the following characteristics: biocompatibility, which means that they should be compatible with the human body; bioresorbability; biodegradability; and appropriate mechanical properties, depending on the implantation site [1,2,3,4].

Biocompatibility is the most often-used phrase to define the proper biological requirements of a biomaterial or biomaterials used in a medical device. Biocompatibility has also been defined as a material’s capacity to operate with an adequate host reaction in a certain application. In contrast, biodegradability is the ability of living organisms to biologically degrade organic materials to their base constituents, such as water, carbon dioxide, methane, the basic elements, and biomass. Living organisms such as bacteria and fungi in the natural environment, ecology, or biomedicine can decompose or break down biodegradable materials into their basic components at an appropriate degradation rate. Bioresorbability, on the other hand, is more sensitive than biodegradability, due to its more specific requirements that the byproducts be biocompatible. Bioresorbable materials are those that can be degraded or dissolved in vivo through a series of metabolic and hydrolytic reactions without causing harm to the human body. On the other hand, the main challenge with bioresorbable materials is that they must operate for a specified time at the desired dissolution rate and then disappear under well-defined conditions. In the same context, bio-inks are materials that are utilized in 3D printing to create engineered or artificial living tissue. These inks are largely made up of the cells that are being utilized, but they are frequently combined with other materials that surround the cells. A bio-ink is a mix of cells and biopolymer gels [1,2,3,4].

Tissue engineering is a field wherein the aforementioned bio-printing technique is increasingly being used. Tissue engineering is a cutting-edge, multidisciplinary field that combines principles of engineering and biology to create structures that perform better biologically. In terms of clinical applications, one of the most important goals of tissue engineering is to overcome the numerous barriers imposed by current treatments, which are primarily based on organ transplants and biomaterial use for implantation [5]. Organ and tissue malfunctions are a major source of concern for human health and well-being. Targeted medical intervention is critical, especially when the human body fails to heal itself using its own mechanisms.

Despite the fact that tissue engineering is a relatively recent field, the idea of tissue substitution was first put forth in the 16th century. Gasparo Tagliacozzi (1546–1599), Professor of Surgery and Anatomy at the University of Bologna, succeeded in building a nose replacement using a forearm flap in “De Custorum Chirurigia per Insitionem,” published in 1597 [6], which can be regarded as the initial or first-documented relevant case. The first clinical use of human cells in this discipline was to produce skin tissue using scaffolds, fibroblasts, or keratinocytes (that would act as a tissue substrate). The regeneration process of rabbit articular surfaces using allograft chondrocytes and collagen gel is also reported in another published work [7]. The literature work “Tissue Engineering” by Langer and Vacanti [8] is regarded as a significant contribution to the advancement of tissue engineering research globally [9].

Three-dimensional bioprinting is vital in tissue engineering, which aims to create functional tissues for use in regenerative medicine and drug testing. Bioprinting can provide patient-specific spatial geometry, controlled microstructures, and the positioning of diverse cell types for the fabrication of tissue engineering scaffolds. Tissue regeneration and reconstruction may open up the prospect of repairing or replacing damaged tissues and organs. Its undeniable contribution to society lies in the fact that its application can lead to shorter waiting times for organ donors and less animal testing, because fabricated tissue engineering items are not dependent on biomaterials or scaffolding that are lacking in native tissues.

The first successful uses of 3D bioprinters to develop the first entirely lab-grown organs are dated to the first ten years of the twenty-first century. In this field, cell therapies, tissue-engineering constructs, and organs for many different parts of the body have been developed; Anthony Atala’s contribution is regarded as fundamental [10]. Additionally, he is credited with creating 3D bioprinters [11,12], and in 2006, he and his team created the first lab-fabricated organ for implantation in humans—a human bladder. The work of Dr. Paolo de Coppi, whose group is developing a tissue-engineered pediatric tracheal replacement, is also crucial in this field [13].

**Figure 1:**
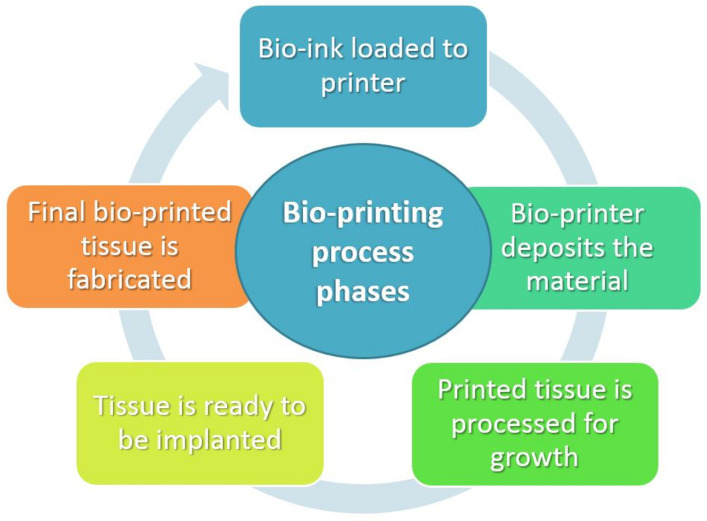
Distinct stages of bio-printing process.

In order to serve as an appropriate substrate for 3D tissue formation, which is the ultimate goal, lattice tissue-engineering constructs created by bioprinters and suitable biomaterials must demonstrate a range of required features. They serve as bioresorbable structures in the area of the defect and are more generally referred to as “scaffolds” due to the fact that they contain cells and hydrogels. They can be classified as “cellular scaffolds” or “acellular scaffolds,” which are scaffolds devoid of cells, such as hip and knee implants, etc. (scaffolds with cells, such as skin constructs). When it comes to maintaining mechanical integrity over periods of healing and degradation, bio-printed scaffolds should display the desired mechanical behavior [14,15]. A connected pore network is thought to be essential for the passage of nutrients and blood under controlled porosity. The ability to fabricate scaffolds in precise geometric shapes is required for the aforementioned reasons [16]. By being able to provide dimensional stability, repeatability, and the production of pre-designed linked porosity networks at different sizes, bio-printing techniques exhibit tremendous potential in this field [17,18]. Cases of bio-printed scaffold structures are discussed in a number of published papers [17,19,20,21,22,23,24,25,26,27,28,29,30,31]. Figure 1 depicts the basic stages of the bio-printing process. Table 1 depicts the raw materials used in bio-printing processes, depending on the modus operandi of each process.

## 2. 3D Bioprinting Techniques

### 2.1. Fused Deposition Modeling (FDM)

The continuous layer-by-layer extrusion of a thermoplastic polymer filament characterizes fused deposition modeling (FDM), a revolutionary scaffold-building technology. The absence of an organic solvent, the quick solidification of the extruded polymer, and the structural integrity of the resulting three-dimensional matrix are all advantages of FDM. The FDM bio-printer’s goal is to fabricate configurable, reproducible scaffolds that facilitate homogeneous cell distribution. Due to its potential to generate regenerative tissues and organs, this technique has paved the way for the development of artificial multicellular tissues and organs. Three-dimensional bioprinting now uses a wide range of biomaterials and different methods developed by researchers.

**Figure 2:**
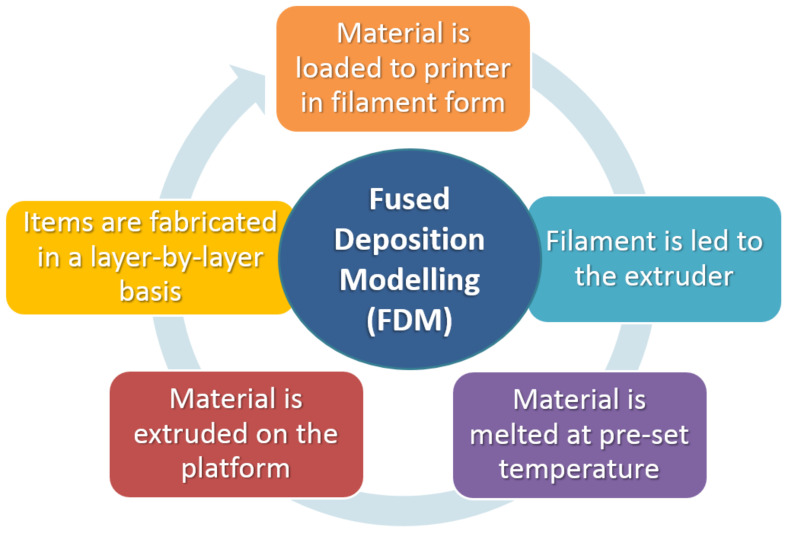
Distinct stages of the fused deposition modelling (FDM) process.

In this method, the material is either melted by the heated nozzle to form a layer on the build platform or it is fed into the extrusion nozzle as a liquid with a predetermined viscosity. The necessary ink is created as a solid filament, which is subsequently heated throughout the extrusion process to a semi-molten condition. The filament material is then oozed out through a temperature-controlled nozzle. Layer by layer, the extruded material that was forced out is deposited onto a platform. The platform is further lowered after one layer is finished, and the subsequent layer is then deposited. Layer thickness or height, printing speed, infill rate, nozzle temperature, retraction, shell thickness, and the potential inclusion of supports (structures that assist deposited materials in being correctly printed in cases of steep angles) are major factors that significantly affect the material’s final qualities [32,33,34,35,36,37,38]. A schematic of a fused deposition modeling (FDM) 3D printer is shown in Figure 2.

Numerous materials can be used with the majority of FDM printers. They must be melted at temperatures between 200 and 250 °C, have a viscosity greater than 6 × 10^7^ mPa/s, and they must solidify quickly in order to melt. Furthermore, less than 5 × 10^5^ s^−1^ should be the value for the elastic-modulus-to-melt-viscosity ratio.

#### Materials Compatible with the FDM Process

**Poly(caprolactone):** PCL is an excellent choice for melt-based extrusion operations because of its characteristics. This material is an attractive option for medical applications because of its thermal stability, low acquisition cost, and shear-thinning capabilities. It is also well-suited for usage in medical devices. According to published literature, PCL may be employed to create a tissue-engineered scaffold that can be used as a substrate for restoring breast tissue after a partial mastectomy procedure [39]. Other cases mentioned in the literature include the design and bio-printing of a novel wound-dressing material by incorporating Juglone (5-hydroxy-1,4-naphthoquinone) to a 25% Polycaprolactone (PCL) scaffold, which demonstrated high rates of targeted wound-healing [40], and the use of a PCL material combined with sodium mesoglycan (MSG), which exhibited high rates of healing [41].

**Poly (lactic acid):** One of the most often-used polymers for FDM is polylactic acid (PLA), which features benefits such as biocompatibility, biodegradability, and affordability. This material’s melting point allows for the formation of filaments, and it can be extruded at temperatures between 180 and 250 °C [42]. When PLA breaks down, acidic byproducts are released, which is one of its problems. The release of lactic acid results in a considerable reduction in physiological acidity. A composite material made of PLA and ceramics is used to lessen the possibility of acidic leakage. The composite material is a promising choice for tissue-engineering operations due to the fact that it also has a tendency to improve the strength of compressive pressures.

**Polyether ether ketone (PEEK):** Peek is a semi-crystalline thermoplastic polymer with a service temperature of 260 °C and a melting point between 330 and 340 °C [9]. It was previously not used in FDM procedures because of its high melting point. PEEK can now be used in FDM printers thanks to recent improvements in printer technology. According to the research that has been published, the melting point temperature, the speed of the extrusion, and the amount of force are the three most important factors that affect the 3D printing of PEEK using FDM procedures [43].

**Poly-vinyl alcohol (PVA):** Vinyl alcohol and acetate monomers combine to form the synthetic polymer known as poly-vinyl alcohol (PVA). These latter qualities—biocompatibility, biodegradability, and bio-inertia—are provided by their presence. PVA can be utilized in filament form in FDM processes, and is soluble in lukewarm water. This substance’s tensile characteristics are quite similar to those of human articular cartilage, making it an ideal substrate for the ingrowth of bone cells [44]. Its semi-crystalline shape permits optimal oxygen and nutrient delivery to the cell, and its hydrophilicity and chemical stability allow for exposure to high pH and temperature conditions. PVA is widely employed in a variety of load-bearing implant applications, including bone-tissue-regeneration procedures and cranio-facial deformities [45,46].

### 2.2. Stereolithography (SLA)

A popular bioprinting technique for meeting the needs of fabricating complex tissues is stereolithography (SLA) 3D bioprinting. The potential of SLA 3D bioprinting in a variety of subsectors, including bone and neural tissue engineering and the creation of controlled microenvironments to study cell behavior, is increased by the development of novel photocrosslinkable biomaterials with improved physical and chemical properties. SLA bioprinting is an innovative bioprinting technology with a wide range of potential and clinical applications thanks to its adaptable design and versatility. Light-based bioprinting, which is an adaptation of stereolithography (SLA) procedures, creates constructions by starting chemical reactions that firm or cure bioinks only where they have been illuminated. Such bioprinters are often faster, using many types of light-based technologies, such as digital light processing (DLP), because they cure full layers concurrently. Light-based bioprinters can also recreate more delicate details at considerably greater resolutions due to millions of microscopic points of light.

A laser or a DLP projector is used in the SLA printing method of 3D printing to layer-by-layer cure a photo-polymer resin. The photosensitive resin layer in a tank of SLA 3D printers is cured using a UV laser or a DLP projector. A very thin, solid coating is created when the resin is cured or hardened by the light source. This slice adheres to the platform or the layer that was previously generated. After this, the build plate starts to deviate from a value that corresponds to the predetermined layer thickness. The fabrication of an item is repeated in this manner until the entire object is produced.

**Figure 3:**
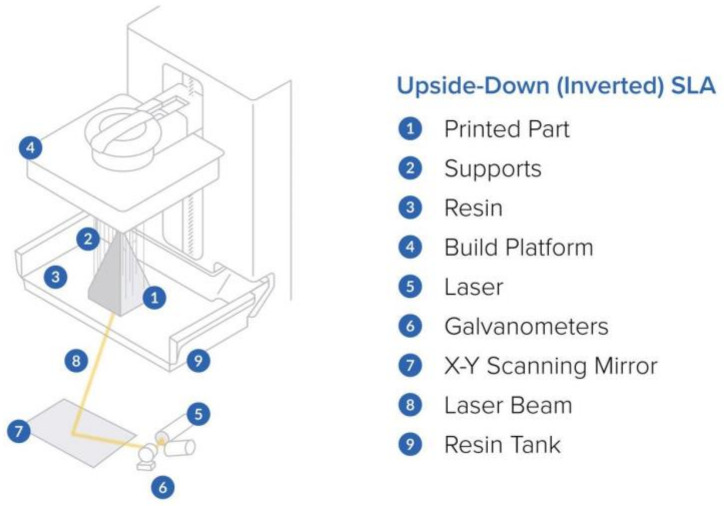
Graphical representation of the basic components in a stereolithography (SLA) 3D printer [48].

Due to the fact that a light source is used to harden the material, SLA printing results in smaller prints and a higher resolution than FDM printing. The size of the light-source spot determines the horizontal resolution of an SLA scanner, which can be anywhere between 30 and 140 microns. The Z-direction resolution, often known as the vertical resolution, ranges from 25 to 200 microns [47]. The layer height and potential support location must be properly configured in order to produce a high-quality print. Figure 3 shows a graphical representation of the basic components in a stereolithography (SLA) 3D printer.

Optical transparency, low viscosity, biocompatibility, and UV stability are all characteristics that ink materials that work with the SLA process should have [49].

#### Materials Compatible with SLA Process

**Poly(D,L-lactide) (PDLLA):** PDLLA is a flexible polymer with numerous medicinal uses, such as scaffolding for tissue engineering, controlled drug delivery, and synthetic nerve conduits constructed of PDLLA, -TCP, and collagen for the regeneration of peripheral nerves [50,51,52]. The fabrication of composite scaffolds from HA biocement embedded in PDLLA oligomers using SLA 3D printing technology is described in the literature. N-methyl-2-pyrrolidone (NMP) is used as a diluent and ethyl 2,4,6-trimethylbenzoylphenylphosphinate acts as a photoinitiator. A non-reactive diluent, such as NMP, is required to help maintain the desired viscosity for SLA, because the viscosity of the resin increases as the percentage of ceramic in the resin increases. It has been discovered that the elasticity of the material rises as the concentration of HA particles increases [53].

**Poly (propylene fumarate) (PPF):** PPF is used in SLA due to it exhibiting pho-to-cross-linkability. It also has superior mechanical properties, and it is biodegradable. In the majority of cases, it is used in SLA by combining diethyl fumarate (DEF) as a solvent with PPF as the base polymer. A photo-initiator is needed in SLA in addition to the previously mentioned fix. Bisacryl phosphrine oxide is utilized in this case. The right amounts of PPF and DEF must be present. Mechanical strength has been seen to significantly decrease at PPF to DEF ratios greater than 0.5 [54]. On the other hand, adding DEF reduces the solution’s viscosity and enhances its printability. PPF molecular mass, viscosity, and molecular mass distribution can now be precisely determined thanks to the recent development of a ring-opening polymerization process. Reduced molecular mass distribution helps the constructed structures to dissolve over time. PPF material is now used in more biomedical applications thanks to newly developed post-polymerization and post-processing functionalization techniques. According to the recent literature, the influence of PPF molecular mass in scaffolds made using the SLA approach was crucial for controlling the degradation pace and bone regeneration in vivo. While there was no documented inflammation or host cell acceptance, PPF with a lesser mass demonstrated finer behavior in healing rates [54].

**PEGDA & GelMA inks**: The application of SLA-based 3D bio-printing for a unique cell-laden cartilage-tissue assembly has been documented in the literature. The resin utilized was made up of transforming growth factor-beta 1 (TGF-1)-embedded nanospheres created using a core-shell electrospraying process, 10% gelatin methacrylate (GelMA) as a base material, and varying percentages of polyethene glycol diacrylate (PEGDA). It was discovered that adding PEGDA to GelMA hydrogel significantly enhanced printability, and that PEGDA also increased compressive modulus correspondingly while decreasing the swelling ratio. The maximum cell viability and proliferation rates were observed in cells cultured on 5%/10% (PEGDA/GelMA) hydrogel. The TGF-1 implanted in nano-spheres can maintain a sustained release for up to 21 days and enhance the encapsulated MSCs’ chondrogenic development. As a result, such materials exhibit high potentials in cartilage-regeneration applications [55,56].

### 2.3. Direct Ink Writing (DIW)

**Figure 4:**
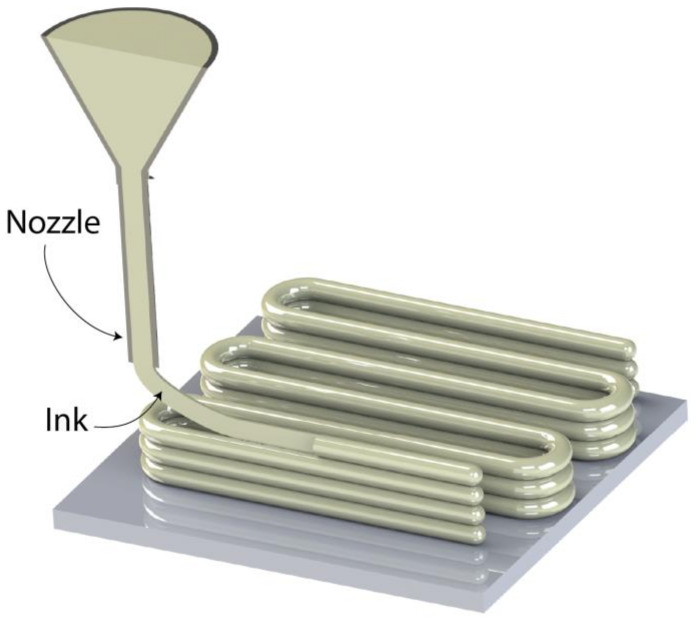
Graphical representation of a direct ink writing 3D printing process [58].

The extrusion-based 3D-printing technique known as DIW uses a nozzle to extrude materials onto a build platform layer-by-layer, similarly to the FDM method. By using this method, it is possible to control the deposition of raw materials in a highly viscous liquid condition, allowing them to keep their shape during the deposition stage. Due to the fact that it can use a wide range of materials, including ceramics, hydrogels, plastic, food, and even living cells, DIW can be seen as being more versatile than FDM. Nozzle size, material viscosity and density, printing speed, and thickness maintained between layers are the main factors that determine the final properties of the fabricated item. Similarly to FDM and SLA, support structures must be used in DIW when complicated geometric shapes with overhangs and steep deposition angles are present. However, the use of dissolvable materials as supports helps to overcome this problem because they can be quickly removed after the printing process is complete. By using UV-curing equipment, the post-fabrication processing phases further aid in improving the printed item’s mechanical characteristics (such as elastic modulus) [57]. Figure 4 depicts a graphical representation of a direct ink writing 3D-printing process

Ink materials used in a DIW method should exhibit fast rates of gelation and maintain proper structural integrity upon the completion of the printing process.

### 2.4. Laser-Guided Direct Writing (LGDW)

LGDW, a laser-assisted direct writing method, can deposit cells with micrometer-level precision. On a number of surfaces and matrices, cell deposition with a focused laser beam is possible. In the process of “laser-guided bio-printing,” cells are guided by a laser beam onto a receiving substrate [59].

#### Materials Suitable for DIW & LGDW

**Hydrogel Inks:** Complex three-dimensional networks of hydrophilic polymers that have a high water-absorption capacity are called hydrogels. Due to their capacity to provide the ideal conditions that favor the encapsulation of viable cells, as well as protecting the cells without obstructing cell–cell communication, these materials have a number of advantages, but their greatest benefit is their high biocompatibility and biodegradability rates. The regulated viscosities of hydrogel inks should enable fluid flow under working pressure conditions. Additionally, they must offer sufficient structural integrity when printing is finished, as well as a quick rate of gelation that can be managed by utilizing shear thinning [58]. According to the literature, creating a polymer solution that solidifies into a network after printing is the preferred method for creating hydrogel ink. Utilizing external stimuli such as temperature, light, or ion concentration, the network thus created could be physically or chemically crossed-linked [60]. The majority of synthetic and natural polymers, including polyacrylamide, polyurethane, and polyethene glycol (PEG), are used in the fabrication of hydrogels in 3D bioprinting [61,62]. Examples of natural polymers used in this process include gelatin, cellulose, collagen, fibrinogen, alginate, and agar.

As the polymer concentration and cross-linking density rise, the rate of proliferation toward the targeted tissue declines. Higher polymer concentrations, however, have been discovered to be ideal for extrusion-based DIW due to their increased viscosity. As the polymer concentration rises, the mechanical properties increase. Shear stress can result in cell death during extrusion because it increases with viscous inks, high pressures, and small diameter nozzles. According to research, shear stresses greater than 60 MPa can result in 35% or more cell death [63]. Shear stress is the parameter that has the greatest impact on resolution in hydrogel bioprinting when using the DIW technique.

**Gelatin-methacryloyl (GelMA):** Using gelatin that has been derivatized with methacrylamide and methacrylate groups, GelMA is a semi-synthetic hydrogel. GelMA hydrogels were demonstrated to support the formation of cartilage tissue using chondrocytes and MSCs in experiments conducted by Sauty et al. [63]. GelMA hydrogels can be synthesized with a specific degree of functionalization (DoF) and tailored to the intended application as a three-dimensional (3D) cell-culture platform. Piao et al. discovered that significantly higher pressure was needed to dispense cell-laden GelMa from glass capillary, but that cell vitality and proliferates weren’t significantly impacted [64,65].

Neural tissues must meet some specific ink material requirements while being bioprinted using DIW. More specifically, brain-cell development, vitality, and cell signaling are impacted by the elastic modulus, or the stiffness of the utilized composite ink matrix. According to research, brain tissues can withstand a stiffness of about 0.5 MPa, which is significantly less than the stiffness of bone or cartilage tissues. Soft hydrogels must therefore have low interfacial tensions to enable cell movement across the tissue implant line. This can be ensured by combining two or more printing inks, one of which will have the necessary biological characteristic and the other of which will have the duty of controlling the stiffness percentage [66]. Nucleic acid delivery and bio-printing can be combined in a number of ways, according to recent research. Among them is one working on gene-activated bioink. In this instance, the target nucleic acid and its delivery method could be combined in a single step by encapsulating them in a bio-printable substance, producing a gene-activated bio-ink [67].

### 2.5. Inkjet Bioprinting

**Figure 5:**
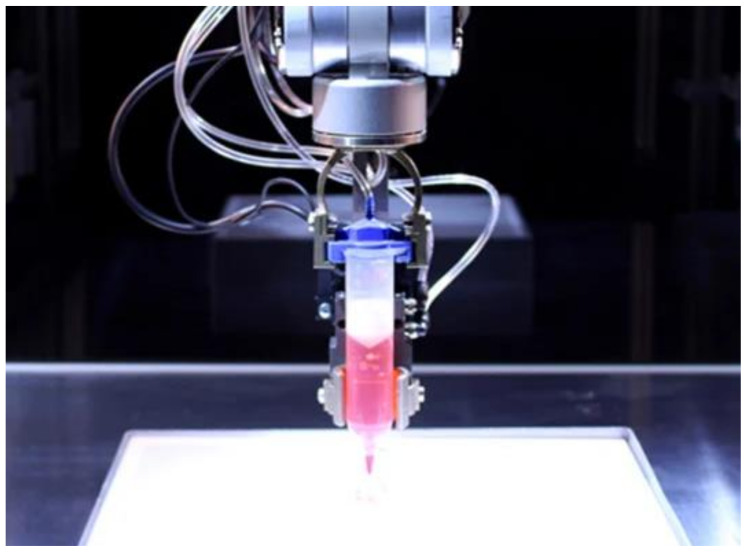
Inkjet 3D bio-printer [70].

A non-contact, controlled 3D-printing technique called inkjet printing enables the dispersion of droplets with volumes ranging from 1 to 100 picoliters that still contain cell viability. One of two methods—continuous inkjet (CI) or drop-by-drop (DOD)—is used to extrude droplets from a nozzle (CIJ). Of the two, DOD is better suited for tissue engineering. In addition, DOD can be divided into three categories based on the depositing method. Some examples include mechanical, electromagnetic, and thermal (which use heat to expand and deposit the material before the nozzle). In DOD inkjet 3D bio-printing, individual drops with diameters ranging from 25 to 50 μm are produced in accordance with predetermined specifications. A continuous stream of individual droplets with a volume of 100 μm in diameter are expelled during CIJ 3D bioprinting. Due to the fact that these techniques have a tendency to influence the cell wall and its vitality after being sonicated at 15–25 Hz, it is discovered that electromagnetic and thermal inkjet printing have not been widely accepted as far as tissue engineering is concerned. Due to this, thermal inkjet is used more frequently to promote better cell vitality [68]. The process is accelerated by the use of many inkjet print heads, each of which has a number of separate nozzles. When using a thermal inkjet printer, the ink is heated to a high temperature, which causes bubbles to form. The bubbles continue to grow until the ink is discharged from the nozzle. The survivability of biologically printed DNA, cells, tissues, and other organs is not affected by the heating temperature, which can reach up to 300 °C for a brief period of time. In this instance, cell viability has been determined to be about 85% [69]. An inkjet 3D bio-printer is shown in Figure 5.

The desired viscosity and surface tension are dictated by the ink requirements in this technique because viscosity affects clogging and surface tension greatly influences the shape of the drop not only after it leaves the nozzle but also on the substrate. The ink’s viscosity should be less than 10 centipoises, and its surface tension should typically fall between 28 and 350 mN m^−1^ [71].

It is worth mentioning that all of the aforementioned materials need to undergo sterilization and disinfection protocols, which are essential in the field of tissue engineering. Every material has its own necessities according to its unique molecular structure, as well the implantation’s site environment. For example, Han et al. [72] used ultrasonic cleaning with deionized (DI) water and 70% ethanol before drying with nitrogen for 20 s, regarding PEEK disc samples. Finally, the PEEK disks were sterilized via autoclave sterilization at 134 °C for 5 min. Told et al., describe the use of various materials and techniques in order to achieve the desirable sterilization and disinfection degrees. These ranged from the use of ethanol in 70% (V/V) concentration, chlorine in the form of chlorine-based tablets containing sodium-dichloroisocyanurate-dihydrat, and techniques such as gas plasma dehydration, autoclaving, and dry-heat sterilization [73]. In addition, Rynio et al., also used heat (105 °C and 121 °C), hydrogen peroxide plasma, and ethylene oxide gas as sterilization and disinfection methods in the case of three-dimensionally printed aortic templates [74].

## 3. Conclusions

Three-dimensional printing technology has advanced significantly over the last decade, with many new processes being developed in a number of disciplines and industries. Reduced fabrication times are now available, as are newly developed materials with a variety of characteristics. The development of 3D bioprinters that use biomaterials compatible with the human body allows for the creation of highly regulated porous, interconnected structures that serve as biological substrates for human cells to proliferate and form tissues. These structures must have a number of properties, including biocompatibility, bioresorbability, and the desired mechanical behavior. In this context, sophisticated biomaterials, such as bio-inks used as raw materials in 3D bio-printers, may now create high viability cells, tissues, and can even directly create DNA. The careful adjustment of process parameters in 3D bio-printer settings, as well as the continuous introduction of novel biomaterials, provide the only feasible method to fully exploit the capabilities of this technology.

## Figures and Tables

**Table 1:** Raw materials used in bio-printing processes.

FDM Process	SLA Process	Direct Ink Writing & Laser-Guided Direct Writing Processes
Polycaprolactone (PCL) Polylactic acid (PLA) Polyether ether ketone (PEEK) Poly-vinyl alcohol (PVA)	Poly(D,L-lactide) (PDLLA) Polypropylene fumarate (PPF) PEGDA & GelMA inks	Hydrogel inks Gelatin-methacryloyl (GelMA)

## Data Availability

Not applicable.
